# Homozygous Pathogenic Variant in BBS9 Gene: A Detailed Case Study of Bardet–Biedl Syndrome

**DOI:** 10.7759/cureus.65774

**Published:** 2024-07-30

**Authors:** Ali A Al-Mat'hammi, Saif A Alzahrani, Fahad Saleh Alsefry, Suhaib Ghurab, Mohammed Alghamdi

**Affiliations:** 1 Pediatric Endocrinology, King Fahad Hospital, Albaha, SAU; 2 Pediatrics, King Fahad Hospital, Albaha, SAU

**Keywords:** setmelanotide, polydactyly, heterogeneity, syndrome, bbs

## Abstract

In this case study, we describe an eight-year-old Saudi girl diagnosed with Bardet-Biedl syndrome (BBS), characterized by a rare homozygous mutation in the *BBS9 *gene. She presented with typical symptoms including obesity, polydactyly, developmental delays, and cognitive difficulties. This case underscores the genetic heterogeneity of BBS and demonstrates the crucial role of comprehensive genetic testing in identifying this complex ciliopathy. It highlights the need for a multidisciplinary strategy to manage the diverse manifestations of BBS, which includes surgical correction of polydactyly and customized educational support. Additionally, we explore the therapeutic possibilities of setmelanotide, an emerging treatment for obesity associated with BBS, highlighting advancements in treatment approaches for genetic disorders. This report adds to the existing knowledge of the genetic variability of BBS and emphasizes the role of personalized medicine in mitigating its extensive clinical effects.

## Introduction

Overview of Bardet-Biedl syndrome (BBS)

In 1866, Laurence and Moon first documented a condition in a family of four siblings exhibiting symptoms of retinal dystrophy, obesity, spastic paraparesis, and intellectual deficits. Subsequently, Bardet and Biedl described additional cases with similar symptoms, including postaxial polydactyly. This led to the condition being named Laurence-Moon-BBS. The condition is now differentiated into Laurence-Moon syndrome and BBS, despite their significant clinical overlap, which suggests a potential genetic link between them. Currently, the term BBS is universally accepted and used [1].

BBS is an inherited disorder characterized by its diverse clinical manifestations. It represents a prototypical ciliopathy, with symptoms resulting from defects in primary cilia, cellular structures essential for signal transduction. BBS is known for its considerable phenotypic variability and genetic heterogeneity, making it a complex condition to study and understand [2].

Genetic and epidemiological aspects

BBS is primarily inherited in an autosomal recessive pattern. More than 20 genes (BBS1-BBS21) associated with this syndrome have been identified, each contributing to ciliary structure and function [2]. The frequency of BBS varies globally, with an estimated prevalence of one in 160,000 in North America and Europe, and it is higher in populations with greater instances of consanguinity.

Pathophysiology

The central pathophysiological feature of BBS is ciliary dysfunction. Cilia are crucial for cellular communication and play critical roles in various signaling pathways during development and in maintaining cellular and tissue homeostasis. Mutations in BBS genes disrupt ciliary function, leading to the wide spectrum of clinical manifestations observed in this syndrome [3].

Clinical manifestations

The clinical presentation of BBS is varied, with primary features including rod-cone dystrophy leading to progressive vision loss, postaxial polydactyly, central obesity developing in childhood, male hypogonadism and female genitourinary malformations, renal abnormalities that can be structural or functional, and mild learning disabilities. Secondary features encompass metabolic abnormalities such as diabetes mellitus and hyperlipidemia, cardiovascular anomalies, hepatic involvement, hearing loss, and dental anomalies [3].

Diagnostic criteria and challenges

Diagnosis of BBS is multifaceted, involving a combination of clinical findings and genetic testing. The Beales criteria, a widely recognized diagnostic guideline, include four primary features (retinal dystrophy, obesity, polydactyly, cognitive impairment, hypogonadism, and genitourinary abnormalities, and renal abnormalities) and secondary features. A definitive diagnosis typically requires the presence of either four primary features or three primary and two secondary features. Genetic testing can confirm the diagnosis and is particularly valuable in atypical cases or when full clinical criteria are not met.

However, diagnosing BBS is challenging because of the overlap with other ciliopathies and the genetic heterogeneity of the syndrome. Additionally, the variable expression of symptoms, even among individuals with the same genetic mutation, adds complexity to the diagnostic process.

Management and prognosis

Management is symptomatic and involves a multidisciplinary approach. Regular ophthalmologic assessments are crucial because of progressive vision loss. Monitoring and management of renal function, metabolic syndrome, and other systemic conditions are essential. Early intervention and supportive therapies can improve the quality of life and prognosis. The life expectancy in BBS varies, with renal disease being a significant determinant.

Current research and future directions

Ongoing research in BBS focuses on a better understanding of the genetic basis and pathophysiology of the disorder. This includes exploring the role of cilia in various cellular processes and identifying potential therapeutic targets. Advances in genetic and molecular technologies offer hope for novel interventions, including gene therapy and personalized medicine approaches.

## Case presentation

We describe the case of an eight-year-old Saudi girl who presented at the pediatric clinic of King Fahad Hospital in Albaha City with obesity and polydactyly concerns. The patient, the firstborn to first-cousin consanguineous parents, had a notable maternal history of two miscarriages related to ectopic and anembryonic gestations and a sibling who died at two months from undetermined causes, no similar case in the family.

**Figure 1:**
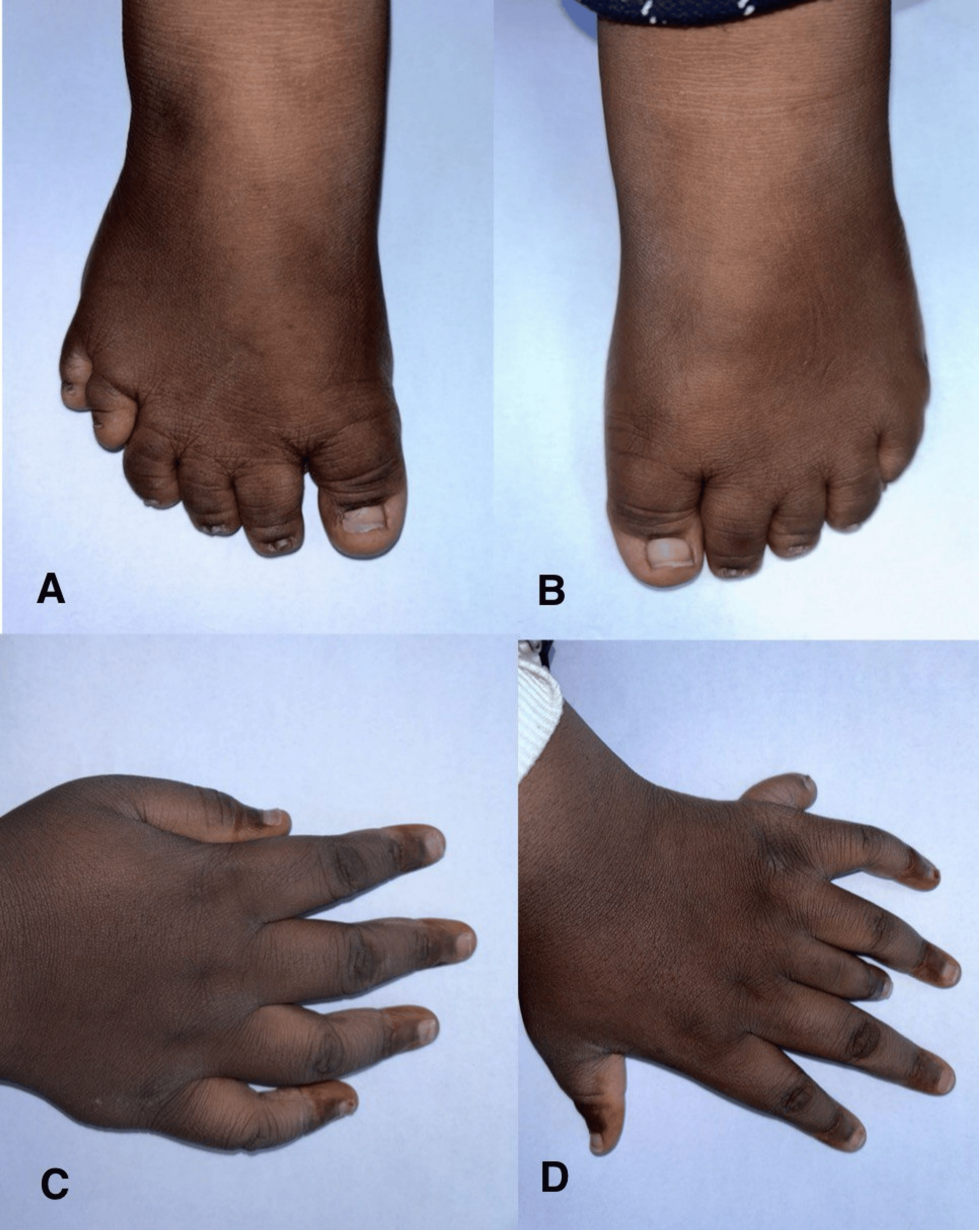
(A) Right foot of the patient showing postaxial polydactyly. (B) Left foot of the patient with the extra digit removed. (C) Right hand of the patient with the extra digit removed. (D) Left hand of the patient showing postaxial and central polydactyly.

No antenatal scans were performed in this case. The girl was born via spontaneous vaginal delivery at full term without any complications during or immediately after delivery. Her birth weight was 2 kg. At birth, physicians noted polydactyly, characterized by an extra postaxial finger on each limb, except for the left hand, which had two additional fingers: which showed a postaxial and central polydactyly (Figures 1A-1D).

Initially, as the child grew, the only observable abnormality was polydactyly. Developmental delays became evident over time. Although she started regular school at seven years of age, cognitive impairments and poor academic performance necessitated withdrawal and led to a recommendation for cognitive testing. Psychiatric assessment identified significant deficits in attention, recognition, processing speed, response time, and verbal and written communication skills. She received diagnoses of moderate intellectual disability and attention deficit disorder, with recommendations for speech therapy and behavioral modification, which were initiated. Notably, there was a significant delay in her speech development. She interacts well with her parents and is generally playful but quiet. While playing with other children, she often displays a reserved demeanor. Additionally, she developed vision issues that required corrective glasses by the age of seven.

Upon her presentation at our clinic at the age of eight years, the patient is morbidly obese, with a weight of 57 kg and a height of 130 cm, giving her a BMI of approximately 33.8, which places her above the 97th percentile for her age according to the Saudi growth charts. She had a history of obstructive sleep apnea, elevated blood pressure, acanthosis nigricans, and nystagmus, complicating her clinical picture. The mental status examination indicated moderate intellectual disability.

Given her clinical presentation, which included several features consistent with the Beales criteria for BBS, a comprehensive diagnostic workup was initiated (Table 1). This workup included a panel of laboratory tests (complete blood count (CBC), liver function tests (LFTs), renal function tests (RFTs), thyroid function tests (TFTs), lipid profile, and cortisol level) that showed elevated HbA1C and low HDL cholesterol levels (Table 2). An abdominal ultrasound revealed no abnormalities. Notably, Whole Exome Sequencing (WES) confirmed a diagnosis of BBS with a homozygous pathogenic variant in the *BBS9* gene.

**Table 1:** Beales criteria for Bardet-Biedl syndrome

Feature	Incidence (4)
Major features	
Retinal cone-rod dystrophy	94%
Central obesity	89%
Postaxial polydactyly	79%
Cognitive impairment	66%
Hypogonadism & genitourinary abnormalities	59%
Kidney disease	52%
Minor features	
Neurologic abnormalities	
- Developmental delay	81%
- Epilepsy	9.6%
- Behavior/psychiatric abnormalities	35%
Olfactory dysfunction	47%–100%
Oral/dental abnormalities	~50%
Cardiovascular & other thoraco-abdominal abnormalities	1.6%–20%
Gastrointestinal abnormalities	
- Hirschsprung disease	2.8%
- Inflammatory bowel disease	1.1%
- Celiac disease	1.5%
- Liver disease	30%
Endocrine/metabolic abnormalities	
- Metabolic syndrome	54.3%
- Subclinical hypothyroidism	19.4%
- Type 2 diabetes mellitus	15.8%
- Polycystic ovary syndrome	14.7%

**Table 2:** Laboratory findings for the patient

Test	Result	Normal Range
Hemoglobin (HB)	12.40 g/dL	13.8 to 17.2 g/dL (men), 12.1 to 15.1 g/dL (women)
White blood cells (WBC)	8 x 10^9^/L	4.5 to 11.0 x 10^9^/L
Red blood cells (RBC)	5.48 x 10^12^/L	4.7 to 6.1 x 10^12^/L (men), 4.2 to 5.4 x 10^12^/L (women)
Mean corpuscular hemoglobin (MCH)	22.70 pg	27.5 to 33.2 pg
Mean corpuscular hemoglobin concentration (MCHC)	31 g/dL	33.4 to 35.5 g/dL
Red cell distribution width (RDW)	15.40%	11.6 to 14.6%
Mean corpuscular volume (MCV)	73.20 fL	80 to 100 fL
Packed cell volume (PCV)	40.10%	38.3 to 48.6% (men), 35.5 to 44.9% (women)
Platelet count	426 x 10^9^/L	150 to 450 x 10^9^/L
Albumin	41.30 g/L	35 to 50 g/L
Alanine transaminase (ALT)	50 U/L	7 to 56 U/L
Aspartate transaminase (AST)	33.12 U/L	0 to 40 U/L
Blood urea nitrogen (BUN)	3.40 mmol/L	2.5 to 7.1 mmol/L (7 to 20 mg/dL)
Creatinine	46 µmol/L	44 to 133 µmol/L (0.5 to 1.5 mg/dL)
Sodium	139 mmol/L	135 to 145 mmol/L
Potassium	4.70 mmol/L	3.5 to 5.0 mmol/L
HB A1c	6.21%	Less than 6.5%
Thyroid-stimulating hormone (TSH)	1.229 mIU/mL	0.7 to 4.0 mIU/mL
Free T4	11.35 pmol/L	9 to 22 pmol/L
Free T3	6.93 pmol/L	3.5 to 7.8 pmol/L
Follicle-stimulating hormone (FSH)	0.75 mIU/mL	Less than 4.0 mIU/mL
luteinizing hormone (LH)	0.42 mIU/mL	Less than 4.0 mIU/mL
Cholesterol	3.07 mmol/L	Less than 4.4 mmol/L (170 mg/dL)
Triglyceride	1.96 mmol/L	Less than 2.32 mmol/L (100 mg/dL)
High-density lipoprotein (HDL)	0.77 mmol/L	Greater than 0.9 mmol/L (35 mg/dL)
Low-density lipoprotein (LDH)	1.90 mmol/L	Less than 2.85 mmol/L (110 mg/dL)

The management of her condition involved consultations with multiple specialties, including ophthalmology, ENT, psychiatry, nutrition, and plastic surgery, to address the wide range of manifestations associated with BBS. Surgical intervention was limited to the removal of extra digits on her right hand and left foot, with plans to address the remaining polydactyly. The interdisciplinary approach also extended to her educational needs, facilitating her transition to a specialized school to support her learning and development better.

Summary of case presentation

An 8-year-old Saudi girl presented with obesity and polydactyly. Born to consanguineous parents, with a history of maternal miscarriages and a sibling who died at two months, she was delivered full term weighing 2 kg. At birth, she exhibited polydactyly with extra fingers on each limb, except for the left hand, which had two additional fingers.

Despite normal speech development, she showed cognitive impairments and poor academic performance, leading to withdrawal from regular school and diagnoses of moderate intellectual disability and attention deficit disorder. By age 8, she was morbidly obese (57 kg, 130 cm) with a BMI above the 97th percentile for her age. Her medical history included obstructive sleep apnea, elevated blood pressure, acanthosis nigricans, and nystagmus.

A comprehensive workup confirmed BBS with a homozygous pathogenic variant in the *BBS9 *gene. Management involved multiple specialties and surgical removal of extra digits, with plans for further surgeries. An interdisciplinary approach was adopted to support her medical and educational needs.

## Discussion

BBS is named after Georges Bardet and Arthur Biedl, who independently described the syndrome in the early 20th century, characterized by obesity, polydactyly, retinal degeneration, and hypogonadism. Initially, this condition was confused with Laurence-Moon syndrome, described earlier by John Zachariah Laurence and Robert Charles Moon, which includes retinal dystrophy and neurological symptoms but lacks the obesity and polydactyly typical of BBS. Consequently, the combined term Laurence-Moon-Biedl-Bardet syndrome has been deprecated in favor of recognizing BBS and Laurence-Moon syndrome as separate entities because of their distinct clinical presentations and genetic backgrounds. While discussions about the overlap between these conditions have occurred, the current consensus maintains their classification as separate entities [4].

BBS is primarily inherited in an autosomal recessive pattern. Over 20 genes associated with this syndrome have been identified, each contributing to ciliary structure and function. The prevalence of BBS varies globally, with an estimated frequency of one in 160,000 in North America and Europe, but it is higher in populations with greater instances of consanguinity [4].

Our report on an eight-year-old girl diagnosed with BBS highlights the complexity of managing this genetically diverse and phenotypically variable condition. The identification of a homozygous pathogenic variant in the *BBS9* gene, which is considered rare, accounted for only 3.4% of all the gene types implicated in BBS [5]. This case underscores the critical role of genetic testing in the diagnostic process of BBS, particularly in cases with a consanguineous background. The genetic findings in our patient contribute to the growing body of literature that supports the diverse genetic landscape of BBS, where more than 20 genes have been implicated. This heterogeneity poses a challenge for diagnosis and management but also opens avenues for personalized medicine approaches [6].

Our case highlights the importance of a multidisciplinary approach to care in BBS, demonstrating the syndrome’s extensive impact on multiple organ systems. The involvement of specialists in ophthalmology, ENT, psychiatry, nutrition, and plastic surgery was crucial in addressing the wide array of manifestations, from polydactyly and obesity to cognitive impairment and vision issues. This comprehensive approach facilitated targeted interventions, such as the surgical removal of extra digits, and supported the patient’s developmental needs through specialized educational services. The necessity and success of such interdisciplinary collaboration underscore the importance of early and accurate diagnosis.

The diagnostic journey of our patient emphasizes the applicability and limitations of the Beales criteria for diagnosing BBS. Although these criteria serve as a valuable guide, the symptomatic overlap with other ciliopathies and the variable expression of BBS symptoms necessitate a meticulous and thorough evaluation. Our experience advocates for the integration of genetic testing as a fundamental component of the diagnostic process, providing a definitive path to diagnosis in complex cases and offering crucial information for family planning and counseling.

The early diagnosis and management of our patient also illuminate the potential benefits of timely intervention, particularly in mitigating some of the syndrome’s progressive aspects, such as vision loss, renal abnormalities specifically associated with BBS9, and cognitive decline. However, the variability in clinical presentation, even among individuals with the same genetic mutation, poses a continual challenge. This variability emphasizes the need for personalized management plans and points to areas for future research, including the investigation of genotype-phenotype correlations and the development of targeted therapies.

Future research should aim to elucidate the pathophysiological mechanisms underlying the diverse manifestations of BBS, with a particular focus on understanding the role of the *BBS9* gene. Such studies are essential for developing targeted interventions that could address the root causes of the syndrome's features, rather than merely managing its symptoms. Additionally, investigating the outcomes of various management strategies on long-term quality of life and disease progression in BBS patients can provide invaluable insights into optimizing care for this complex condition.

Weight management is an important aspect to be addressed in BBS patients, considering its potential complications in children as they age. In 2022, setmelanotide (Imcivree) received FDA approval for chronic weight management in patients aged 6 years and older with BBS, marking a significant advancement in the treatment options available for this condition. This approval positions setmelanotide as the first drug specifically approved for chronic weight management in BBS patients. The drug is also indicated for obesity-related to proopiomelanocortin (POMC), proprotein convertase subtilisin/kexin type 1 (PCSK1), or leptin receptor (LEPR) deficiency.

The efficacy of setmelanotide was assessed in a 66-week study involving 44 patients diagnosed with BBS and obesity. This study comprised a 14-week, randomized, double-blind, placebo-controlled phase followed by a 52-week open-label period. Results indicated that patients receiving setmelanotide achieved an average reduction of 7.9% in their body mass index (BMI) after 52 weeks. Furthermore, 61% of patients experienced a reduction of 5% or more in their BMI, and 39% saw a reduction of 10% or more​​ [7].

Setmelanotide is accompanied by specific warnings and precautions. It can cause disturbances in sexual arousal, including spontaneous penile erections in males, and is associated with risks of suicidal ideation and depression, which necessitates monitoring for mood alterations and suicidal thoughts. Additionally, there have been observations of increased skin pigmentation and alterations in pre-existing moles, making skin examinations before and during treatment essential. The most frequent adverse reactions are skin hyperpigmentation, injection site reactions, gastrointestinal symptoms (nausea, vomiting, diarrhea), and spontaneous penile erection​​​​.

The approval of this drug marks a significant advancement for the BBS community by providing a new strategy for weight management and addressing one of the syndrome’s most daunting challenges. Setmelanotide functions by circumventing disruptions in leptin signaling to the brain, which causes an unrelenting urge to eat in individuals with BBS​​.

## Conclusions

In summary, our case study contributes to the broader understanding of BBS, emphasizing the complexities and achievements in diagnosing and managing this genetically and phenotypically diverse condition. They highlight the critical role of genetic testing in confirming the diagnosis and the benefits of a multidisciplinary approach to delivering comprehensive care. Our research supports the need for heightened awareness and early diagnostic initiatives to enhance prognosis and life quality for patients with BBS.
